# Körperliche Aktivität und psychische Gesundheit im Kindes- und Jugendalter: Zusammenfassung der Evidenz aus 3 nationalen Studien

**DOI:** 10.1007/s00103-026-04312-9

**Published:** 2026-10-20

**Authors:** Franziska Reiß, Claudia Niessner, Anne Kaman, Carmen Volk, Steven Behn, Ann-Kathrin Napp, Anke Hanssen-Doose, Alexander Woll, Ulrike Ravens-Sieberer

**Affiliations:** 1 Zentrum für Psychosoziale Medizin, Klinik für Kinder- und Jugendpsychiatrie, -psychotherapie und -psychosomatik, Forschungssektion „Child Public Health“, Universitätsklinikum Hamburg-Eppendorf, Hamburg, Deutschland; 2 Institut für Sport und Sportwissenschaft, Karlsruher Institut für Technologie, Karlsruhe, Deutschland; 3 Institut für Bewegungserziehung und Sport, Pädagogische Hochschule Karlsruhe , Karlsruhe, Deutschland

**Keywords:** Bewegungsverhalten, Psychische Auffälligkeiten, Gesundheitsbezogene Lebensqualität, Soziale Ungleichheit, Bevölkerungsstudien, Movement behaviour, Mental health problems, Health-related quality of life, Social inequality, Population-based studies

## Abstract

Heranwachsende fühlen sich durch globale Krisen zunehmend belastet. Körperliche Aktivität (KA) gilt als bedeutsamer Baustein für die mentale Gesundheit, jedoch fehlt für Deutschland eine integrierte Zusammenschau bevölkerungsbasierter Evidenz. Ziel dieser narrativen Übersicht ist es daher, aktuelle Befunde zum Zusammenhang zwischen KA und psychischer Gesundheit zusammenzuführen.

Grundlage bilden 3 bevölkerungsbezogene Studien, welche als Modul- bzw. Anschlussstudien in Kooperation der Studie zur Gesundheit von Kindern und Jugendlichen in Deutschland (KiGGS) durchgeführt wurden: die Motorik-Modulstudie (MoMo), die BEfragung zum seeLischen WohLbefinden und VerhAlten (BELLA), die Child Outcomes in PSYchology (COPSY)-Studie sowie die COMO-Studie (COPSY + MoMo, interdisziplinäre Verknüpfung). Untersucht wurden bundesweit repräsentative Stichproben von Kindern und Jugendlichen im Alter von 4–23 Jahren unter Einsatz validierter Instrumente zur Erfassung von KA und psychischer Gesundheit. Die Ergebnisse werden narrativ zusammengeführt und differenziert nach Aktivitätsform, Alter, Geschlecht und sozialem Status berichtet.

Es zeigen sich positive Zusammenhänge zwischen KA und psychischer Gesundheit. Höhere Aktivität geht mit geringeren psychischen Auffälligkeiten und höherer gesundheitsbezogener Lebensqualität einher. Besonders für Sport in der Freizeit im Verein und außerhalb des Vereins zeigen sich Hinweise auf protektive Effekte. Selbstberichtete KA weist stärkere Zusammenhänge mit Wohlbefinden auf als KA, die gerätegestützt erfasst wurde. Kinder aus sozioökonomisch benachteiligten Familien sind seltener aktiv und stärker belastet. Als weitere zentrale psychosoziale Faktoren erweisen sich die soziale Einbindung sowie das Kompetenzerleben im Sport. KA kann eine wichtige Ressource zur Förderung der psychischen Gesundheit darstellen. Es zeigt sich die Notwendigkeit, niederschwellige Bewegungsangebote zu fördern, die insbesondere benachteiligte Gruppen erreichen, um gesundheitliche Ungleichheiten abzubauen.

## Abstract (en)

Young people are increasingly burdened by global crises. Physical activity (PA) is considered a key factor for mental health; however, for Germany, an integrated synthesis of population-based evidence is still lacking. The aim of this narrative overview is therefore to consolidate current findings on the association between PA and mental health.

The analysis draws on three population-based studies conducted as module or follow-up studies in cooperation with the KiGGS study: the MoMo study (motor performance/physical activity), the BELLA and COPSY studies (mental health), and the COMO study (interdisciplinary integration). Nationwide representative samples of children and adolescents aged 4 to 23 years were examined using validated instruments to assess PA and mental health. Findings are synthesised narratively and reported by type of activity, age, sex and socioeconomic status.

Overall, positive associations between PA and mental health are observed. Higher levels of PA are associated with fewer psychological problems and higher health-related quality of life. There is evidence of protective effects, particularly in relation to leisure-time sport, both within and outside sports clubs. Self-reported PA shows stronger associations with well-being than objectively measured PA. Children from socioeconomically disadvantaged families are less likely to be physically active and experience more mental health problems. Social integration and sense of competence in sport have been identified as further key psychosocial factors. PA can therefore be considered an important resource for promoting mental health. There is a need to promote low-threshold access to physical activity programmes that specifically target disadvantaged groups in order to reduce health inequalities.

## Einleitung

Das Aufwachsen von Kindern und Jugendlichen wird wesentlich durch das Gesundheitsverhalten beeinflusst, wobei insbesondere das Bewegungsverhalten eine zentrale Bedeutung für die gesundheitliche Entwicklung hat. Nach dem Verständnis der Weltgesundheitsorganisation (WHO) wird Gesundheit zudem nicht allein als körperliche Gesundheit verstanden, sondern schließt ebenso psychisches und soziales Wohlbefinden ein [1]. Internationale Metaanalysen zeigen, dass die psychische Gesundheit von Kindern und Jugendlichen in den vergangenen Jahren durch die COVID-19-Pandemie und weitere globale Krisen stark beeinträchtigt worden ist [2, 3]. Die Lancet Psychiatry Commission on Youth Mental Health spricht in diesem Zusammenhang sogar von einer globalen Krise der psychischen Gesundheit junger Menschen und fordert eine stärkere Priorisierung von Prävention und Frühintervention [4]. Vor diesem Hintergrund ist es eine zentrale Frage der Public-Health-Forschung, welche Faktoren die psychische Gesundheit von Kindern und Jugendlichen stärken. Körperliche Aktivität gilt dabei als ein vielversprechender Ansatzpunkt.

Ein theoretisches Rahmenmodell zur Erfassung des Zusammenhangs von körperlicher Aktivität und psychischer Gesundheit – als zentrale Elemente des gesunden Aufwachsens im Kindes- und Jugendalter – wird von Lubans et al. [5] in ihrem systematischen Review vorgestellt. Dieses Modell ist wegweisend, da es diesen Zusammenhang über 4 miteinander verknüpfte Komponenten strukturiert. Die Verbindung der körperlichen Aktivität mit der psychischen Gesundheit hängt demnach von verschiedenen Merkmalen der Aktivität selbst ab – darunter Häufigkeit, Intensität, Dauer, Art und Kontext. Individuelle Merkmale wie Alter, Geschlecht und früheres Bewegungsverhalten beeinflussen diesen Zusammenhang zusätzlich. Zudem verdeutlicht das Modell, dass der Zusammenhang über verschiedene neurobiologische, psychosoziale und verhaltensbezogene Faktoren erklärt werden kann. Auf Ebene der Gesundheitsindikatoren wird zwischen kognitiven Funktionen, Indikatoren des Wohlbefindens und psychischer Belastung im Sinne internalisierender und externalisierender Probleme differenziert. Eine gute psychische Gesundheit ist demnach auch das Ergebnis dieser unterschiedlichen Faktoren, die gerade bei jungen Menschen besonders stark ineinandergreifen, da sich das Gehirn und die Identität noch in einer kritischen Entwicklungsphase befinden [5]. White et al. [6] identifizierten in ihrem umfassenden systematischen Review eine starke Evidenz für psychosoziale Mediatoren (z. B. Selbstwirksamkeit) sowie moderate Evidenz für Geschlecht und Alter als Moderatoren [6]. Die empirische Überprüfung dieser Wirkzusammenhänge steht jedoch insbesondere für bevölkerungsbasierte Stichproben von Kindern und Jugendlichen noch aus.

Mit Blick auf die aktuelle internationale Studienlage wird deutlich, dass höhere körperliche Aktivität mit geringeren psychischen Auffälligkeiten verbunden ist, wobei der Zusammenhang bei Jugendlichen im Vergleich zu jüngeren Kindern signifikant stärker ist [7]. Differenzierte Analysen für spezifische psychische Störungsbilder fanden vergleichbare Effekte sowohl für internalisierende als auch für externalisierende Auffälligkeiten [8]. Auch bei der gesundheitsbezogenen Lebensqualität – als ein positiv konnotierter Indikator psychischer Gesundheit – zeigen sich zwar kleine, aber konsistent positive Effekte körperlicher Aktivität auf das Wohlbefinden [9], die durch neuere Arbeiten bestätigt wurden [10].

Während die internationale Evidenz somit zunehmend differenzierte Antworten auf die Frage nach dem „Ob“ und „Wie“ des Zusammenhangs liefert, ist die Datenlage für Deutschland auf Bevölkerungsebene noch nicht zusammenfassend dargestellt. Mit der Gesundheitsberichterstattung des Robert Koch-Instituts (RKI) assoziierte bevölkerungsbasierte Studien oder deren Nachfolgestudien tragen zu einem differenzierten Bild bei. Zudem liefert auch die Move For Health-Studie [11] für 2023 repräsentative Daten zur sportlichen Aktivität und mentalen Gesundheit von Jugendlichen und fand schwache, aber signifikante Zusammenhänge zwischen körperlicher Aktivität bzw. Sportvereinsmitgliedschaft und psychosozialer Gesundheit. Soziale Ungleichheitsmerkmale (z. B. Bildungsstand der Eltern) beeinflussten sowohl das Aktivitätsverhalten als auch die Gesundheit [11].

Ziel dieses Beitrags ist, die aktuelle Evidenz zum Zusammenhang zwischen körperlicher Aktivität und psychischer Gesundheit bei Kindern und Jugendlichen in Deutschland darzustellen. Aufbauend auf dem von Lubans et al. [5] entwickelten Modell sowie der Datenbasis dreier RKI-assoziierter bevölkerungsbasierter Studien werden publizierte Befunde zu folgenden Fragestellungen narrativ zusammengefasst: (1) Zeigt sich ein Zusammenhang zwischen der körperlichen Aktivität und der psychischen Gesundheit (psychische Auffälligkeiten, gesundheitsbezogene Lebensqualität) von Kindern und Jugendlichen in Deutschland? (2) Gibt es Unterschiede in Bezug auf verschiedene Formen der körperlichen Aktivität, z. B. Sport in der Freizeit im Verein und außerhalb des Vereins? (dabei werden sowohl selbstberichtete als auch gerätegestützte Aktivitätsmaße einbezogen)? (3) Zeigen sich unterschiedliche Assoziationen von körperlicher Aktivität mit der psychischen Gesundheit in Abhängigkeit von Geschlecht, Alter und familiärem sozioökonomischen Status?

## Datengrundlage

**Abb. 1:**
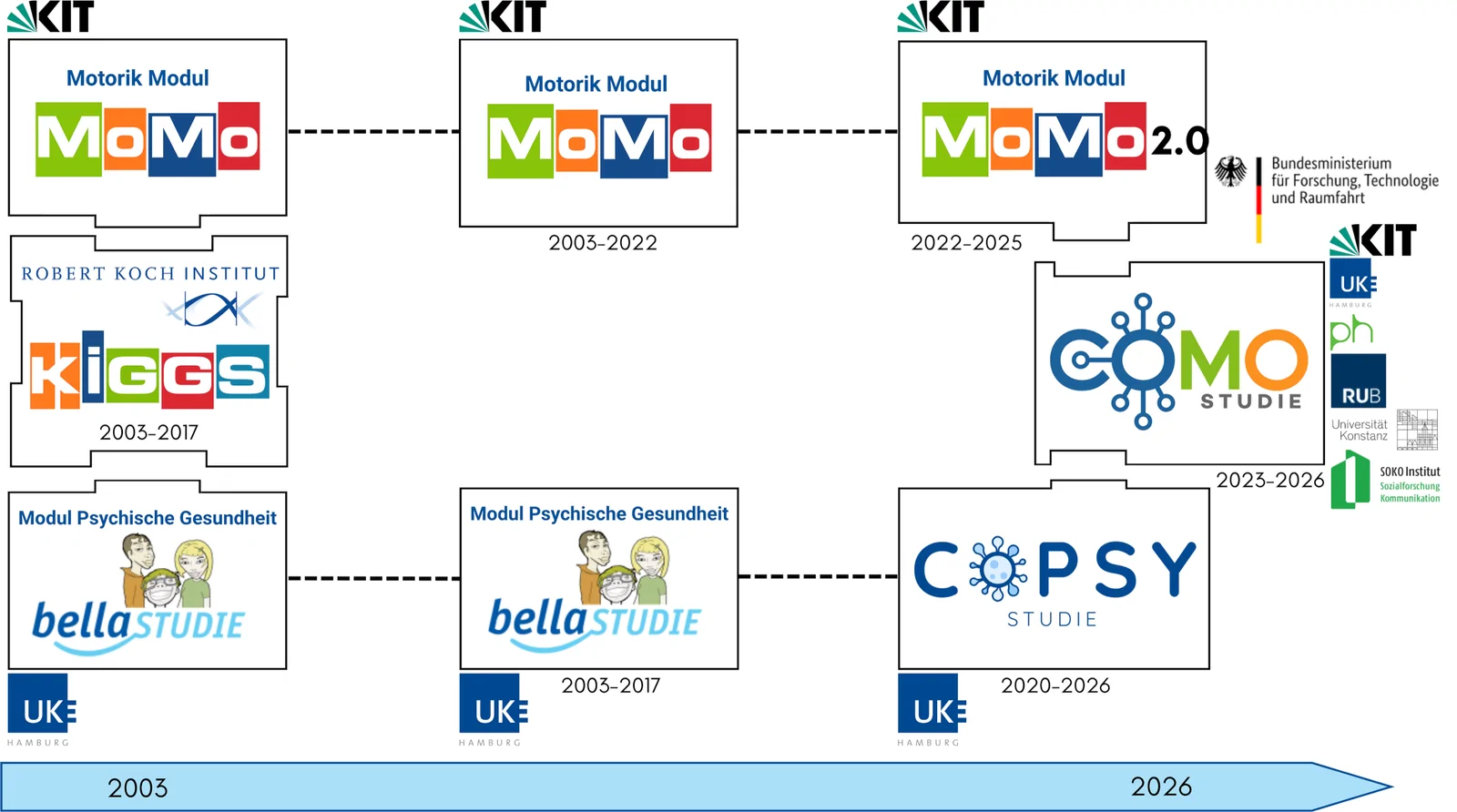
Darstellung der Entwicklung der Studie zur Gesundheit von Kindern und Jugendlichen in Deutschland (KiGGS), Motorik-Modulstudie (MoMo), BEfragung zum seeLischen WohLbefinden und VerhAlten (BELLA), Child Outcomes in PSYchology (COPSY), COPSY + MoMo (COMO) im zeitlichen Verlauf. *KiGGS* Studie zur Gesundheit von Kindern und Jugendlichen in Deutschland; *MoMo* Motorik-Modulstudie; *BELLA* BEfragung zum seeLischen WohLbefinden und VerhAlten; *COPSY* Child Outcomes in PSYchology; *COMO* COPSY + MoMo

Der vorliegende Beitrag verfolgt einen narrativen Übersichtsansatz. Die eingeschlossenen Publikationen stammen ausschließlich aus den 3 bevölkerungsbasierten Studien, die als Modulstudien der *Studie zur Gesundheit von Kindern und Jugendlichen in Deutschland* (KiGGS) bzw. als deren Folgestudien eine gemeinsame methodische und stichprobenbezogene Grundlage teilen. Ein Überblick über die in diesem Beitrag verwendeten Studien findet sich in Abb. 1 sowie Angaben zum Studiendesign und zur Stichprobenzusammensetzung in Tab. 1.

**Tab. 1:** Studiendesign und Stichprobenzusammensetzung der MoMo‑, COPSY- und COMO-Studie

Beschreibung MoMo-Studie	Studiendesign	Repräsentative bundesweite Längsschnittstudie (Kohorten-Sequenzdesign; [12, 13])
	Start	KiGGS-Basiserhebung 2003
	Stichprobe	*N* = 4528 (4–17 Jahre) bei Basiserhebung 2003–2006
	Ziel	Entwicklung motorischer Leistungsfähigkeit und körperlicher Aktivität sowie deren Determinanten und Auswirkungen auf körperliche und psychische Gesundheit
	Stichprobenziehung	167 bundesweite Untersuchungsorte (regionale Repräsentativität)
	Erhebungswellen	T1: 2009–2012; T2: 2014–2017; T3: 2018–2022
	Stichprobenumfang	T1: *N* = 5104; T2: *N* = 6233; T3: *N* = 4175
	Kontext	COVID-Ergänzung „MoMo Snapshots“: 04/2020, 02/2021, 02/2023
	Aktuelle Welle	MoMo 2.0 T4: 2023–2024; *N* = 5825
	Besonderheit	Kombination von Längsschnitt- und Kohortenvergleichen; Analyse langfristiger Trends (> 20 Jahre) und Kriseneffekte
Beschreibung COPSY-Studie	Studiendesign	Bevölkerungsbasierte Längsschnittstudie zur psychischen Gesundheit
	Start	Frühjahr 2020 (COVID-19-Pandemie)
	Stichprobe	*N* = 1586 bei Basiserhebung 05–06/2020
	Ziel	Untersuchung von psychischer Gesundheit und Wohlbefinden über verschiedene Krisenphasen
	Stichprobenziehung	Panel-Stichprobe
	Erhebungswellen	T1: Mai–Juni 2020; T2: Dez. 2020–Jan. 2021; T3: Sep.–Okt. 2021; T4: Feb. 2022; T5: Sep.–Okt. 2022; T6: Okt.–Nov. 2023; T7: Okt. 2024; T8: Sep.–Okt. 2025
	Stichprobenumfang	T1: *N* = 1586; T2 *N* = 1625; T3: *N* = 1618; T4: *N* = 1668; T5: *N* = 1701; T6: *N* = 1672; T7: *N* = 1505 Gesamt: *N* = 3312 Familien (mind. eine Welle), Alter 7–23 Jahre
	Kontext	Abbildung verschiedener Krisenphasen (Lockdown, Lockerungen, Ukraine-Krieg, Energiekrise, Post-Pandemie); Referenz: BELLA-Studie 2014–2017 (vergleichbare Methodik [14])
	Aktuelle Welle	COPSY T8: Sep.–Okt. 2025; *N* = 1607
	Besonderheit	Wiederholte Befragung + Auffrischungsstichproben zur Sicherung der Repräsentativität; Online-Befragung; Elternberichte für Kinder ab 7 Jahren + Selbstberichte ab 11 Jahren
Beschreibung COMO-Studie	Studiendesign	Repräsentative bevölkerungsbasierte Längsschnittstudie
	Start	Oktober 2023
	Stichprobe	COMO 1 *N* = 5240 (4–17 Jahre)
	Ziel	Körperliche Aktivität und psychische Gesundheit systematisch zu verknüpfen und deren Zusammenspiel im Kontext der Pandemie und darüber hinaus umfassend zu untersuchen unter Einbeziehung sozioökologischer Kontexte
	Stichprobenziehung	Ziehung aus einer bundesweiten Registerstichprobe (Einwohnermeldeamtsstichprobe, 2‑stufiger Ziehungsprozess)
	Erhebungswellen	W1: Okt. 2023–Feb. 2024; W2: 2024–2025; W3: 2025–2026
	Stichprobenumfang	T1: *N* = 5240; T2: *N* = 2663
	Kontext	Weiterentwicklung und Verknüpfung der Studien zu körperlicher Aktivität und psychischer Gesundheit von Kindern und Jugendlichen
	Aktuelle Welle	COMO T3: 2025–2026; *N* = 2741
	Besonderheit	Interdisziplinäres und multizentrisches Verbundvorhaben

*KiGGs* Studie zur Gesundheit von Kindern und Jugendlichen in Deutschland, *MoMo* Motorik-Modulstudie, *BELLA* BEfragung zum seeLischen WohLbefinden und VerhAlten, *COPSY* Child Outcomes in PSYchology, *COMO* COPSY + MoMo, *COVID-19* Coronavirus Disease 2019 *T* Messzeitpunkt, *N* Fallzahl

Die *Motorik-Modulstudie (MoMo)* sowie die *BEfragung zum seeLischen WohLbefinden und VerhAlten (BELLA)* wurden ursprünglich als Vertiefungsstudien der KiGGS-Studie konzipiert und ermöglichten eine differenzierte Erfassung der körperlichen Aktivität sowie der psychischen Gesundheit von Kindern und Jugendlichen in Deutschland. Während der COVID-19-Pandemie wurden beide Studien unabhängig von der KiGGS-Studie als eigenständige Längsschnittstudien weitergeführt: die psychische Gesundheit im Rahmen der BELLA-Folgestudie *Child Outcomes in PSYchology (COPSY)* und die körperliche Aktivität in der MoMo-Studie. Diese parallelen Studien lieferten wichtige, jedoch getrennte Erkenntnisse zu den Auswirkungen der Pandemie auf unterschiedliche Gesundheitsdimensionen. Vor dem Hintergrund des Bedarfs, Zusammenhänge zwischen körperlicher Aktivität und psychischer Gesundheit auf Gruppen- und Individualebene analysieren zu können, wurde ergänzend die *COMO-Studie (****CO****PSY+****Mo****Mo)* initiiert. Für weitere Informationen zum Design der COMO-Studie siehe Niessner et al. [15, 16].

Die relevanten Publikationen wurden anhand von Veröffentlichungen aus den jeweiligen Forschungsprojekten zusammengestellt. Die Auswahl orientierte sich an den thematischen Schwerpunkten der Fragestellungen und den festgelegten Gesundheitsindikatoren. Ergebnisse wurden nicht aus den Primärdaten, sondern aus den publizierten Einzelveröffentlichungen extrahiert. Tab. 2 schlüsselt die Befunde nach Einzelpublikationen in Bezug auf die jeweils zugrunde liegende Stichprobe, Erhebungszeitraum, Operationalisierung und Analysemethodik auf. Im Folgenden werden die Studiendesigns, Stichproben und relevanten Erhebungsinstrumente beschrieben.

**Tab. 2:** Übersicht der eingeschlossenen Studien und Publikationen

Studie	Publikation	Zeitraum	Design	*N*	Alter	Körperliche Aktivität (KA)	Psychische Gesundheit	Statistische Analyse	Zentrale Befunde
MoMo	Spengler et al. [17]	2003–2006	Querschnitt	1828	11–17 J.	MoMo-Fragebogen: Sport in der Freizeit im und außerhalb des Vereins, Schule (min/Woche), Bildung eines Gesamtindex [12]	KINDL‑R (Gesamtscore)	Lineare und multiple Regression	Gesamtindex (Sport in der Freizeit in und außerhalb des Vereins, Sport in der Schule): Signifikanter Prädiktor der gesundheitsbezogenen Lebensqualität; Zusammenhang blieb nach Kontrolle für Alter, Geschlecht, sozioökonomischen Status und Wohnumfeld bestehen. Ergebnisse des einfachen linearen Regressionsmodells: (B = 0,007; β = 0,155; *p* < 0,001; adj. R^2^ = 0,024; keine adjustierten Koeffizienten berichtet). Sport in der Freizeit im Verein: Signifikanter Prädiktor im adjustierten multiplen Regressionsmodell (Kontrolle für Alter, Geschlecht, sozioökonomischen Status und Wohnumfeld: β = 0,110; *p* = 0,008). Sport in der Freizeit außerhalb des Vereins: nichtlinearer Zusammenhang im hierarchischen Regressionsmodell (keine Kontrolle zusätzlicher Kovariaten berichtet; quadratischer Term: β = −0,360; *p* = 0,002)
MoMo	Nigg et al. [18]	2003–2006, 2009–2012, 2014–2017	Längsschnitt (3 Wellen, 11 Jahre)	686	5,6–16,9 J.	MoMo-Fragebogen: Tage/normale Woche ≥ 60 min MVPA (WHO 2010)	SDQ, Bildschirmzeit (TV, PC)*	Geschlechts-spezifische Pfadmodelle	Höhere Bildschirmzeit bei Mädchen → schlechtere psychische Gesundheit; psychische Auffälligkeiten bei Jungen → mehr Bildschirmzeit; KA nimmt über die Messzeitpunkte ab (4,87 → 3,29 Tage/Woche); SDQ-Gesamtscore steigt (Mädchen: 7,03 → 11,53; Jungen: 7,79 → 10,11)
MoMo	Wunsch et al. [19]	2014–2017	Querschnitt	1565	M = 14,4 J.	MoMo-Fragebogen (selbstberichtet), Bildung eines Gesamtindex [12], Sport in der Freizeit im Verein und außerhalb des Vereins, Schule (min/Woche: Ø 287,6 min/Woche), 7-Tage-Akzelerometrie (Ø 307,9 min/Woche)	KIDSCREEN-27 (5 Dimensionen), KIDSCREEN-10 (M = 52,15)	Korrelationen, hierarchische Regression	Selbstberichtete KA assoziiert mit fast allen gLQ-Dimensionen (körperliches Wohlbefinden: r = 0,266; Gesamt-GLQ: r = 0,095); objektiv gemessene KA nur mit körperlichem (r = 0,210) und psychischem Wohlbefinden (r = 0,068); objektiv gemessene KA erklärt nur 0,7 % zusätzliche Varianz
MoMo	Wunsch et al. [20]	2018–2020 + April 2020	Längsschnitt (2 Wellen, prä-/intra-COVID)	1711	4–17 J. (M = 10,4)	WHO-Empfehlung 2010, Tage/normale Woche ≥ 60 min MVPA	KIDSCREEN-10 (T-Werte; prä-COVID 44, intra-COVID 41)	Cross-lagged Panel-Modelle	gLQ vor COVID sagte KA während COVID voraus (β = 0,07; *p* = 0,003), v. a. bei jüngeren Kindern und Mädchen; höhere Bildschirmzeit vor COVID → weniger KA während COVID (β = −0,21; *p* < 0,001); weder KA noch Bildschirmzeit sagten gLQ während COVID voraus
COPSY	Behn et al. [21]	2020–2023 (6 Wellen)	Längsschnitt	1819	11–21 J.	KA: Tage/Woche ≥ 60 min MVPA (Gruppierung in 3 Gruppen: niedrig, mittel, hoch)	KIDSCREEN-10 (T-Werte), SDQ (Gesamtproblemwert +4 Subskalen)	Lineare Mehrebenenmodelle	Hohe KA → bessere gLQ (d = 0,38) und weniger Auffälligkeiten (d = −0,20); mittlere vs. niedrige KA mit größerem Unterschied als hohe vs. mittlere; stärkste Effekte für emotionale Symptome (d = −0,10 bis −0,18) und Peer-Probleme (d = −0,14 bis −0,19); signifikante Altersinteraktion: stärkere Zusammenhänge mit psychischen Auffälligkeiten bei älteren Jugendlichen; stabil über Pandemiephasen; keine Moderation durch Geschlecht oder SÖS
COMO	Reiß et al. [22]	2023	Querschnitt	5240	4–17 J.	WHO-Empfehlung 2010 (≥ 60 min/Tag, letzten 7 Tage; Gruppen: erfüllt/nicht erfüllt) + Domänen: Sport in der Freizeit im Verein (Ja/Nein), Sport in der Freizeit außerhalb des Vereins (Ja/Nein)	KIDSCREEN-10 (T-Werte), SDQ (Gesamtproblemwert)	Logistische Regression	WHO-Empfehlung erreicht → weniger Auffälligkeiten (OR = 0,66–0,74 je nach Aktivitätsmaß) und höhere gLQ (OR = 1,19–1,73 je nach Aktivitätsmaß); Verein und Freizeit unabhängig positiv assoziiert mit Outcomes; Zusammenhänge kontrolliert für Alter, Geschlecht, SÖS

*KA* körperliche Aktivität, *MVPA* moderate bis anstrengende körperliche Aktivität, *gLQ* gesundheitsbezogene Lebensqualität, *SDQ* Strengths and Difficulties Questionnaire, *WHO* World Health Organisation (dt.: Weltgesundheitsorganisation), *MoMo-PAQ* MoMo Physical Activity Questionnaire, *OR* Odds Ratio, *SÖS* sozioökonomischer Status, *β* standardisierter Regressionskoeffizient, *d* Cohens d, *r* Pearson-Korrelationskoeffizient, *R*^*2*^ Determinationskoeffizient

*In Nigg et al. [18] wird die Bildschirmzeit nicht als Indikator psychischer Gesundheit, sondern als verhaltensbezogene Variable im längsschnittlichen Pfadmodell zwischen körperlicher Aktivität und psychischer Gesundheit (SDQ) modelliert (s. Ergebnisse)

### Messinstrumente

#### Körperliche Aktivität

Die *körperliche Aktivität* wurde in der MoMo- und COMO-Studie mit dem validierten MoMo-Aktivitätsfragebogen erfasst [12, 23], der Art, Häufigkeit und Dauer der körperlichen Aktivität in verschiedenen Settings abbildet (z. B. Sport in der Freizeit im Verein und außerhalb des Vereins) sowie die Gesamtaktivität ermittelt. Zur Erfassung der Gesamtaktivität von moderater bis intensiver körperlicher Aktivität wurden die Kinder und Jugendlichen gefragt, an wie vielen der letzten 7 Tage bzw. einer normalen Woche sie mindestens 60 min körperlich aktiv waren. Körperliche Aktivität ist definiert als eine Tätigkeit mit erhöhter Herzfrequenz und Atmung [12]. In die Auswertung gingen entweder die Anzahl an Tagen pro Woche (0–7) ein oder es wurden Kategorien gebildet, um z. B. die *WHO-Bewegungsempfehlung *[24] abzubilden („Empfehlung nicht erfüllt“ (0–6 Tage körperlich aktiv) vs. „Empfehlung erfüllt“ (7 Tage körperlich aktiv)).

Das Ausmaß an *sportlicher Aktivität im Verein und außerhalb des Vereins* wurde kategorial (Teilnahme „Ja“/„Nein“), als Sportminutenindex (Minuten pro Woche Sport im bzw. außerhalb des Vereins) oder als Gesamtminutenindex (kurz: Gesamtindex, d. h. Minuten pro Woche als Summe aus Sport in der Freizeit im Verein, außerhalb des Vereins und in der Schule) ausgewertet [12, 17, 19].

Ergänzend wurde körperliche Aktivität in der MoMo-Studie seit Welle 2 (2009) mittels *Beschleunigungssensoren* über 7 Tage gerätegestützt erfasst [25], u. a. zur Bestimmung der Aktivitätsminuten und der Erfüllung der WHO-Bewegungsempfehlungen (ActiGraph, activPAL).

#### Psychische Gesundheit

Die psychische Gesundheit von Kindern und Jugendlichen kann über unterschiedliche Indikatoren erfasst werden. Für den vorliegenden Beitrag wurde eine gezielte Auswahl getroffen. Die Fokussierung auf psychische Auffälligkeiten und die gesundheitsbezogene Lebensqualität in diesem Beitrag erfolgte, weil (1) beide Konstrukte zentrale und international etablierte Kernindikatoren psychischer Gesundheit in dieser Altersgruppe sind, (2) sie komplementäre Dimensionen psychischer Gesundheit abbilden (Beeinträchtigung vs. Wohlbefinden) und (3) sie in großen populationsbasierten Studien valide, reliabel und praktikabel messbar sind.^1^

*Psychische Auffälligkeiten* wurden mit dem Strengths and Difficulties Questionnaire (SDQ) erfasst [26, 27]. Der Gesamtproblemwert setzt sich aus 4 Subskalen zusammen (20 Items), wobei höhere Werte mehr Auffälligkeiten anzeigen. Es wurde zwischen internalisierenden (z. B. emotionale Probleme) und externalisierenden Auffälligkeiten (z. B. Verhaltensprobleme) unterschieden. Anhand etablierter Cut-offs [26, 27] erfolgte eine Einteilung in unauffällig, grenzwertig und auffällig, u. a. für Odds Ratios in der COMO-Studie. Der SDQ wurde je nach Alter als Elternbericht für Kinder und Jugendliche im Alter von 4 bis 10 Jahren sowie als Selbstbericht ab 11 Jahren eingesetzt (COPSY-, MoMo- und COMO-Studie).

*Die gesundheitsbezogene Lebensqualität* wurde mit dem KINDL‑R und KIDSCREEN erfasst. Der KINDL‑R (24 Items) bildet 6 Dimensionen ab und wird auf 0–100 skaliert [28]. Der KIDSCREEN-27 erfasst 5 Dimensionen, der KIDSCREEN-10 liefert einen globalen Index [29]. Die Werte werden per Rasch-Modellierung in T‑Werte transformiert (M = 50, SD = 10; [30]). In der COMO-Studie wurden zusätzlich Kategorien auf Basis deutscher Normdaten gebildet, u. a. zur Berechnung von Odds Ratios [31]. Alle Instrumente nutzen 5‑stufige Antwortskalen und sind international validiert [31].

Detaillierte Beschreibungen der Methodik und eingesetzten Instrumente finden sich in bisherigen Publikationen der COPSY-Studie [21, 32], der MoMo-Studie [18] sowie der COMO-Studie [15, 16].

## Ergebnisse

Die Ergebnisse der inkludierten Studien zeigen, dass ein Zusammenhang zwischen körperlicher Aktivität, psychischen Auffälligkeiten und der gesundheitsbezogenen Lebensqualität von Kindern und Jugendlichen in Deutschland besteht [z. B. 18, 20–22]. Ergebnisse der COPSY-Studie zeigen: Ein höheres Maß an körperlicher Aktivität (gemessen in Tagen pro Woche mit mindestens 60 min moderater bis intensiver Aktivität, z. B. Laufen, Radfahren) ist konsistent mit weniger psychischen Auffälligkeiten assoziiert. Im Vergleich zu einer geringen Aktivität (0–2 Tage/Woche) geht bereits ein mittleres (3–5 Tage) bis hohes Aktivitätsniveau (6–7 Tage) mit einer signifikanten Reduktion der psychischen Auffälligkeiten einher. Im direkten Vergleich zwischen mittlerer und hoher Aktivität (6–7 Tage/Woche) fiel die Reduktion der psychischen Auffälligkeiten jedoch deutlich geringer aus [21]. Besonders ausgeprägt sind diese positiven Effekte bei der Reduzierung von emotionalen Symptomen, Problemen mit Gleichaltrigen sowie Hyperaktivität und Unaufmerksamkeit. Die MoMo-Studie liefert hierzu wichtige Längsschnittdaten über 11 Jahre (2003–2017): Sie zeigt, dass körperliche Aktivität in der Kindheit geringere Bildschirmzeiten (TV, PC) im Jugendalter vorhersagt, was wiederum indirekt vor psychischen Belastungen schützt, da hohe Bildschirmzeiten besonders bei Mädchen mit verstärkten psychischen Herausforderungen korrelieren [18]. Die aktuelle COMO-Studie bestätigt diese Befunde auch für die Zeit nach der Pandemie: Kinder, die in ihrer Freizeit im Verein und außerhalb des Vereins sportlich aktiv sind, weisen ein deutlich geringeres Risiko für psychische Auffälligkeiten auf (Odds Ratio zwischen 0,66 und 0,74 je nach Aktivitätsmaß [22]). Der Zusammenhang zwischen körperlicher Aktivität und gesundheitsbezogener Lebensqualität wird durch die Studien ebenfalls gestützt. In der COPSY-Studie korrelierte eine höhere körperliche Aktivität über 6 Untersuchungswellen (2020–2023) hinweg mit einer besseren Lebensqualität, wobei die Effekte als klein bis moderat eingestuft wurden [21]. Auch die Ergebnisse der COMO-Studie aus der Datenerhebung 2023 verdeutlichen, dass körperlich aktive Heranwachsende im Vergleich zu inaktiven Gleichaltrigen eine signifikant höhere Chance auf eine gute Lebensqualität haben (Odds Ratio zwischen 1,19 und 1,73 je nach Aktivitätsmaß [22]).

Differenzierte Analysen im Rahmen der MoMo-Studie verdeutlichen, dass das für die Aktivität gewählte Erhebungsverfahren (z. B. Selbstbericht vs. gerätegestützt erfasste Aktivitätsmaße) entscheidend dafür ist, wie stark der Zusammenhang mit der wahrgenommenen Lebensqualität ist. So steht die selbstberichtete körperliche Aktivität in Zusammenhang mit fast allen Dimensionen der gesundheitsbezogenen Lebensqualität, einschließlich des psychischen Wohlbefindens, der sozialen Unterstützung durch Gleichaltrige und der Wahrnehmung des schulischen Umfelds. Demgegenüber zeigt die gerätegestützte Erfassung körperlicher Aktivität (mittels Akzelerometrie) im Vergleich zu selbstberichteten Aktivitätsmaßen geringere Zusammenhänge mit dem Wohlbefinden und erweist sich als weniger konsistent, d. h., dass sie in ihrer Stärke und Richtung je nach Gesundheitsindikator, Operationalisierung und Stichprobenmerkmalen variieren und teilweise nicht signifikant sind [19]. Dies deutet darauf hin, dass die psychologischen und sozialen Vorteile des Sports, wie das Erleben von Kompetenz, sozialer Einbindung im Verein und die Selbstwahrnehmung als „aktive Person“, für die subjektive Lebensqualität wichtiger sein könnten als die reine physische Bewegungszeit. Vor diesem Hintergrund plädiert eine aktuelle Übersichtsarbeit dafür, Bewegungsempfehlungen insbesondere im Hinblick auf die psychische Gesundheit und das Wohlbefinden nicht allein an quantitativen Kenngrößen (Umfang, Intensität, Häufigkeit) auszurichten, sondern Kontext und Qualität der Aktivität stärker einzubeziehen [33]. Zudem zeigt die MoMo-Studie, dass die Lebensqualität vor der Pandemie ein Prädiktor für das Aktivitätsniveau während der Pandemie war, insbesondere bei jüngeren Kindern und Mädchen [20].

**Abb. 2:**
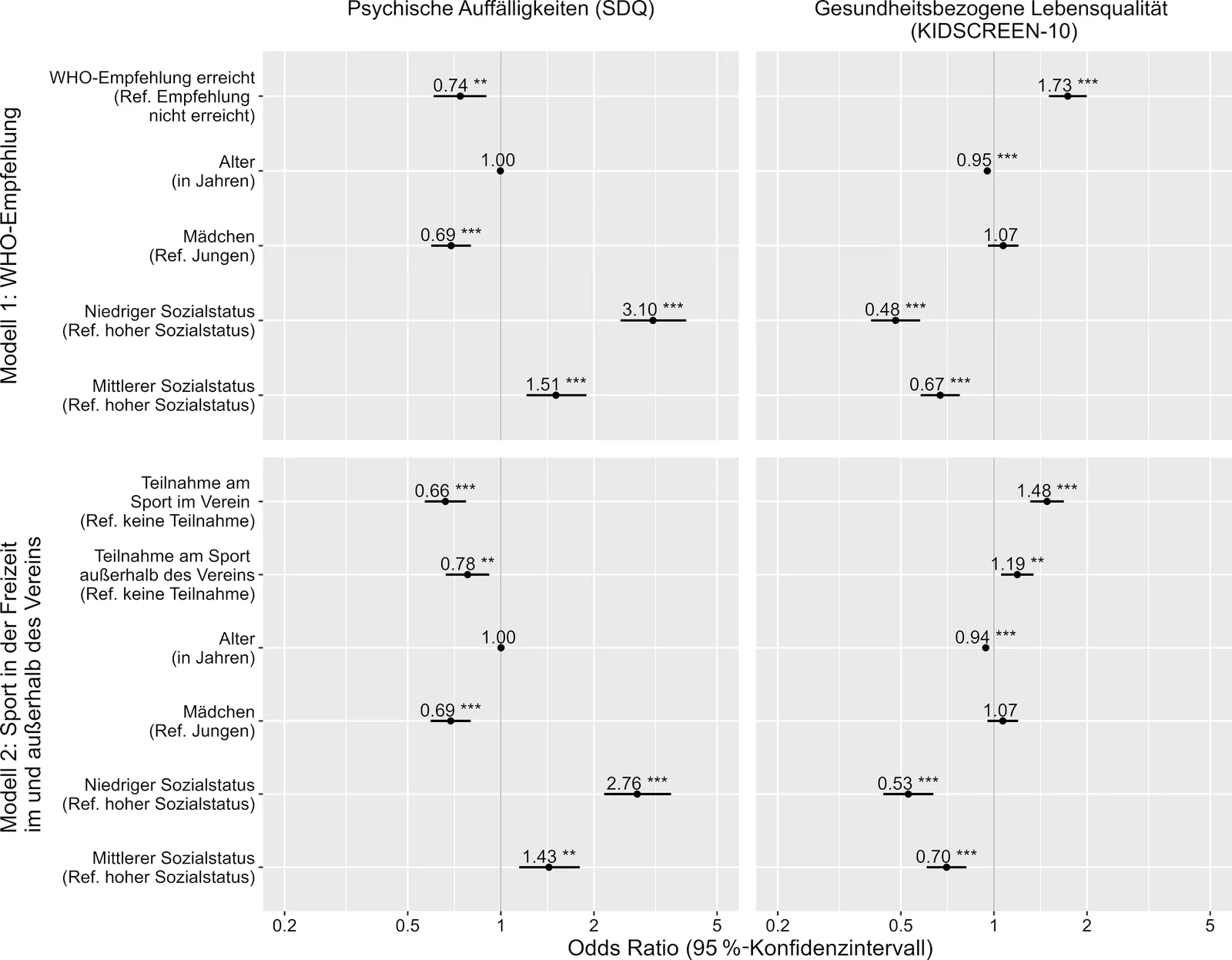
Ergebnisse der logistischen Regression für psychische Auffälligkeiten (SDQ) und gesundheitsbezogene Lebensqualität (KIDSCREEN-10) von Kindern und Jugendlichen. ***p < 0,001, **p < 0,01, *p < 0,05; Modell 1: Zusammenhang zwischen der Erreichung der WHO-Empfehlung (2010) und psychischer Gesundheit *n* = 5125 bzw. gesundheitsbezogener Lebensqualität *n* = 5051; Modell 2: Zusammenhang zwischen der Teilnahme am Sport in der Freizeit im und außerhalb des Vereins und psychischer Gesundheit *n* = 5065 bzw. gesundheitsbezogener Lebensqualität *n* = 4992 [22]. *SDQ* Strengths and Difficulties Questionnaire; *WHO* engl. World Health Organisation (dt.: Weltgesundheitsorganisation); *Ref.* Referenzgruppe

Im Hinblick auf die *zweite Fragestellung* des Beitrags zeigen sich differenzierte, aber insgesamt konsistente Muster hinsichtlich der unterschiedlichen Aktivitätsformen. Für den Vereinssport berichten sowohl die MoMo-, als auch die COMO-Studie, positive Zusammenhänge mit der psychischen Gesundheit und der Lebensqualität [17, 22]. Insbesondere Ergebnisse aus der MoMo-Basiserhebung deuten darauf hin, dass Aktivität im Sportverein ein vergleichsweise starker Prädiktor für die gesundheitsbezogene Lebensqualität ist [17]. Gleichzeitig zeigen die COMO-Befunde, dass auch die Teilnahme am Sport in der Freizeit außerhalb des Vereins mit einer besseren psychischen Gesundheit assoziiert ist [22], welche in Abb. 2 dargestellt sind. Diese Ergebnisse werden durch MoMo-Befunde ergänzt, die für Sport außerhalb des Vereins ebenfalls auf einen positiven, jedoch nicht eindeutig linearen Zusammenhang mit der gesundheitsbezogenen Lebensqualität hinweisen: Während sich in linearen Modellen kein signifikanter Zusammenhang zeigte, deuteten nichtlineare Analysen darauf hin, dass die Lebensqualität zunächst mit zunehmender Freizeitaktivität anstieg und anschließend ein Plateau erreichte [17]. Insgesamt verdeutlichen die Analysen, dass körperliche Aktivität unabhängig von der betrachteten Form mit einer besseren psychischen Gesundheit assoziiert ist, sich jedoch Unterschiede in der Stärke und Art der Zusammenhänge zeigen.

Mit Blick auf *die dritte Fragestellung* zeigen sich geschlechts- und altersspezifische sowie soziökonomische Unterschiede (z. B. Bildung und Einkommen der Eltern) im Zusammenhang mit der körperlichen Aktivität und der psychischen Gesundheit von Kindern und Jugendlichen in Deutschland. In der COMO-Studie zeigten etwa 21 % der Heranwachsenden Hinweise auf psychische Auffälligkeiten, wobei Mädchen häufiger internalisierende Probleme berichten, während bei Jungen eher externalisierende Auffälligkeiten auftreten [22]. Wunsch et al. [20] finden im Rahmen der MoMo-Studie deutliche geschlechts- und altersbezogene Unterschiede im Aktivitätsverhalten, wobei Mädchen sowohl bei den selbstberichteten als auch den gerätegestützten Messungen der körperlichen Aktivität geringere Aktivitätsniveaus aufwiesen als bei Jungen [20]. Diese geschlechtsspezifischen Entwicklungen unterstreichen, dass insbesondere Mädchen stärker von altersbedingten Veränderungen gesundheitsrelevanter Verhaltensweisen betroffen sind und potenziell stärker von einer Steigerung körperlicher Aktivität profitieren könnten [20].

Ergebnisse der COPSY-Studie verdeutlichen, dass Zusammenhänge zwischen körperlicher Aktivität und psychischer Gesundheit im Jugendalter teilweise stärker ausgeprägt sind als im Kindesalter [21]. Befunde der COMO-Studie beschreiben zudem sozioökonomische Unterschiede: Kinder aus Familien mit niedrigem sozioökonomischen Status sind seltener sportlich aktiv (insbesondere im Verein) und leiden gleichzeitig etwa 3‑mal häufiger unter psychischen Auffälligkeiten als Kinder aus statushohen Familien (29,9 % vs. 10,7 % [22]). Dennoch ist körperliche Aktivität unabhängig von Geschlecht und sozioökonomischem Status konsistent mit einer besseren psychischen Gesundheit assoziiert, da entsprechende Zusammenhänge auch nach Kontrolle dieser Faktoren bestehen bleiben [22]. Insgesamt verdeutlichen die Befunde, dass geschlechts- und altersspezifische sowie sozioökonomische Unterschiede zentrale Einflussfaktoren darstellen, die bei der Interpretation der Zusammenhänge zwischen körperlicher Aktivität und psychischer Gesundheit berücksichtigt werden müssen.

Eine Übersicht über die eingeschlossenen Studien ist in Tab. 2 dargestellt.

## Diskussion

Die vorliegenden Befunde aus 3 Studien verdeutlichen, dass der Zusammenhang zwischen körperlicher Aktivität und psychischer Gesundheit bei Kindern und Jugendlichen nicht als linearer oder eindimensionaler Wirkmechanismus interpretiert werden kann [17, 18, 20–22]. Vielmehr sprechen die Ergebnisse dafür, dass beide in ein komplexes Geflecht aus verhaltensbezogenen, psychosozialen und strukturellen Einflussfaktoren eingebettet sind [17, 18, 20, 22]. Diese Erkenntnisse stehen in Einklang mit dem Modell von Lubans et al. [5], das die Zusammenhänge zwischen körperlicher Aktivität und psychischer Gesundheit über Effektmoderatoren (u. a. Art und Kontext der Aktivität) und neurobiologische, psychosoziale und verhaltensbezogene Mediatoren strukturiert ist. Gleichzeitig zeigen die Ergebnisse, dass ein höheres Aktivitätsniveau, unabhängig von der Operationalisierung, mit besserer psychischer Gesundheit und höherer gesundheitsbezogener Lebensqualität assoziiert ist [17, 21, 22]. Die Befunde legen nahe, dass insbesondere der Übergang von einer geringen zu einer mittleren körperlichen Aktivität mit einer verbesserten psychischen Gesundheit verbunden ist. Der zusätzliche Nutzen einer weiteren Steigerung auf ein sehr hohes Aktivitätsniveau fällt demgegenüber geringer aus [21]. Aus Public-Health-Perspektive unterstreicht dies die Bedeutung niedrigschwelliger Maßnahmen, die insbesondere inaktive Kinder und Jugendliche zu mehr Bewegung motivieren, da bereits moderate Aktivitätssteigerungen mit relevanten gesundheitlichen Vorteilen verbunden sein können. Dieser Befund deckt sich mit internationaler Literatur, die ebenfalls ein moderates Aktivitätsniveau bei Erwachsenen mit einer besseren psychischen Gesundheit in Verbindung brachte [34]. Die COPSY-Ergebnisse erweitern diesen Befund auf die Altersgruppe der Kinder und Jugendlichen und unterstreichen, dass der Übergang von körperlicher Inaktivität zu einem moderat aktiveren Lebensstil bereits mit Vorteilen für die psychische Gesundheit einhergehen kann [21].

Unterschiedliche Formen körperlicher Aktivität, sowohl organisierter Sport im Verein, als auch nichtorganisierter Sport außerhalb des Vereins als Freizeitaktivität, leisten eigenständige Beiträge zur psychischen Gesundheit und unterstreichen deren Bedeutung als wichtiger, wenn auch nicht alleiniger Schutzfaktor [17, 22].

Ein zentrales Ergebnis ist die Relevanz der Aktivitätsformen. Die Ergebnisse aus den Studien verdeutlichen, dass nicht nur „wie viel“, sondern insbesondere „wie“ und „wo“ körperliche Aktivität stattfindet, entscheidend ist [17, 22]. Organisierte, sozial eingebettete Aktivitäten zeigen konsistentere Zusammenhänge mit psychosozialen Gesundheitsindikatoren, was die Bedeutung sozialer Integration und strukturierter Interaktionsräume unterstreicht. Diese Ergebnisse decken sich mit internationaler Forschung, die zeigt, dass nicht nur Häufigkeit und Intensität, sondern auch der Kontext entscheidend ist. Teychenne et al. [35] berichten unterschiedliche Effektstärken je nach Aktivitätsdomäne, mit den stärksten Zusammenhängen für Freizeitaktivität (r = 0,21), gefolgt von aktiver Fortbewegung (r = 0,14) und Haushaltstätigkeiten (r = 0,10 [35]). Auch innerhalb der Freizeitaktivität variieren Effekte je nach Sportart und Dimension des Wohlbefindens [36]. Weitere aktuelle Reviews unterstreichen die Rolle organisierter Aktivität für das Wohlbefinden und die Entwicklung von Heranwachsenden [33, 37–39]. Diese Befundlage korrespondiert mit den im Modell von Lubans et al. [5] postulierten Moderatoren Art und Kontext der Aktivität. Entscheidend scheint dabei weniger die organisatorische Rahmung als solche zu sein, sondern vielmehr die mit ihr verbundenen kontextuellen Merkmale, wie beispielsweise die soziale Einbindung, Anleitung und ein strukturiertes Setting, welche den Zusammenhang zwischen körperlicher Aktivität und psychischer Gesundheit günstig beeinflussen können [33, 38]. Gleichzeitig zeigen die vorliegenden Ergebnisse, dass auch nichtorganisierte Aktivität einen eigenständigen Beitrag leisten kann und stärker berücksichtigt werden sollte [33, 38]. Die COPSY-Studienergebnisse legen nahe, dass bereits moderate Aktivitätsniveaus mit besseren Gesundheitsindikatoren assoziiert sind und die Bedeutung niedrigschwelliger Bewegungsförderung unterstreichen [21].

Darüber hinaus variieren die Zusammenhänge in Abhängigkeit vom jeweiligen Kontext. Unterschiede nach Geschlecht und Alter zeigen, dass sich sowohl Bewegungsverhalten als auch dessen gesundheitliche Bedeutung im Entwicklungsverlauf verändern. Insbesondere der Rückgang körperlicher Aktivität und der gesundheitsbezogenen Lebensqualität im Jugendalter, vor allem bei Mädchen, weist auf kritische Entwicklungsphasen hin [20]. Gleichzeitig verdeutlichen sozioökonomische Unterschiede im Zugang zu den Aktivitätsformen, insbesondere im organisierten Sport im Verein, ungleiche gesundheitliche Chancen [22], die eng mit strukturellen Rahmenbedingungen verknüpft sind. Auch in internationalen Reviews finden sich strukturelle Faktoren, wie finanzielle Aspekte, als Barrieren für die Teilnahme am organisierten Sport [40, 41]. Ansätze zur Bewegungs- und Gesundheitsförderung sollten daher explizit soziale und gesundheitliche Ungleichheiten gleichermaßen adressieren und somit allen Heranwachsenden einen Zugang zu körperlicher Aktivität ermöglichen [42]. Darüber hinaus ist zu klären, ob soziale Unterschiedsmerkmale den Zusammenhang zwischen körperlicher Aktivität und psychischer Gesundheit über den ungleichen Zugang hinaus auch in seiner Stärke moderieren. Bislang wurde dies in den vorliegenden deutschen Studien ausschließlich in der COPSY-Studie geprüft, in der sich keine Moderation durch Geschlecht oder sozioökonomischen Status, jedoch eine signifikante Altersmoderation zeigte [21]. Eine systematische Untersuchung solcher Moderationseffekte über verschiedene Aktivitätsformen und Outcomes hinweg steht noch aus und stellt eine wichtige Erweiterung für künftige Studien dar.

Da die vorliegenden Analysen auf Beobachtungsstudiendesigns basieren, sind die Befunde primär als Assoziationen zu interpretieren. Ein Teil der Befunde basiert zudem auf dichotomisierten Aktivitätsmaßen, die den Aktivitätsgradienten auf kategoriale Kontraste reduzieren. Gerätegestützt erhobene Aktivitätsdaten, wie sie in der MoMo-Studie vorliegen, ermöglichen hier differenziertere Auswertungen, etwa nach spezifischen Intensitäts- und Schwellenbereichen. Aussagen zu kausalen Wirkmechanismen bleiben eingeschränkt, insbesondere aufgrund möglicher wechselseitiger Beziehungen sowie Drittvariablen wie sozioökonomischem Status oder familiären Kontextfaktoren. Die Ergebnisse sollten zudem vor dem Hintergrund möglicher Selektionsprozesse interpretiert werden, da die Teilnahme am organisierten Sport im Verein nicht zufällig erfolgt, sondern mit individuellen Ressourcen und psychosozialen Voraussetzungen zusammenhängt [22]. Ein weiterer Aspekt betrifft methodische Unterschiede zwischen den Studien. Trotz der Übereinstimmungen im Studiendesign bestehen Unterschiede in der Erfassung bzw. dem Erhebungsmodus der körperlichen Aktivität [18, 20, 22]. Auch die Analysemethoden variieren (z. B. Quer- vs. Längsschnitt). Diese Heterogenität kann Unterschiede in Effektstärken erklären, liefert jedoch zugleich komplementäre Perspektiven. Einschränkend ist zudem zu berücksichtigen, dass die eingeschlossenen Studien die WHO-Bewegungsempfehlung von 2010 (mindestens 60 min moderate bis intensive Aktivität an 7 Tagen pro Woche) zugrunde legen. Die aktualisierten Empfehlungen von 2020 [43] formulieren stattdessen einen Wochendurchschnitt von 60 min pro Tag, was die Einordnung der hier berichteten Schwellenwerte beeinflusst. Künftige Analysen auf Basis gerätegestützter Aktivitätsdaten können hierzu präzisere Vergleiche liefern. Befunde sind daher stets im Kontext der jeweiligen Methodik zu interpretieren.

Aus Public-Health-Perspektive ergeben sich mehrere Implikationen. Bewegungsförderung sollte Verhalten und Verhältnisse adressieren und neben dem Aktivitätsumfang verstärkt Qualität, Kontext und Zugänglichkeit berücksichtigen. Zudem erfordern soziale Ungleichheiten gezielte Maßnahmen zur Reduktion struktureller Barrieren, die sich an den Bedarfen der Zielgruppen ausrichten. Dafür ist ein multisektoraler Ansatz unter Einbezug von Schule, Kommune und Familie sinnvoll. Gleichzeitig bleibt die Ableitung konkreter Interventionseffekte begrenzt, sodass zukünftige Forschung verstärkt experimentelle und interventionsbasierte Designs berücksichtigen sollte. Insgesamt zeigen die Ergebnisse, dass die Förderung körperlicher Aktivität zur Stärkung der psychischen Gesundheit einen integrierten, kontextsensitiven Ansatz erfordert, der individuelle, soziale und strukturelle Faktoren berücksichtigt.

## Fazit

Der Zusammenhang zwischen körperlicher Aktivität und psychischer Gesundheit bei Kindern und Jugendlichen ist ein komplexes Gefüge aus verhaltensbezogenen, psychosozialen und strukturellen Faktoren. Körperliche Aktivität – auch moderate Bewegung – stellt eine zentrale Ressource dar, die mit weniger psychischen Auffälligkeiten und einer höheren gesundheitsbezogenen Lebensqualität assoziiert ist. Dabei sind vor allem Art und Setting entscheidend: Besonders starke Zusammenhänge mit dem Wohlbefinden zeigen sich für sozial eingebettete, organisierte Angebote. Da die Bewegung im Jugendalter, insbesondere bei Mädchen, spürbar abnimmt und sozioökonomische Zugangsbarrieren zu Sportangeboten bestehen, muss der Zugang gezielt für benachteiligte Gruppen verbessert werden, um gesundheitliche Ungleichheiten abzubauen. Dies erfordert niedrigschwellige, verhältnisorientierte Lösungsansätze über eine multisektorale Zusammenarbeit von Schule, Kommune und Familie.

## Data Availability

Für diesen Artikel wurden keine Datensätze erstellt oder analysiert.

## Footnotes

1 Die Reduktion auf diese beiden Indikatoren dient somit der analytischen Fokussierung und erhöht gleichzeitig die Vergleichbarkeit mit bestehenden Studien und Monitoring-Systemen.
